# Treatment with IL-7 Prevents the Decline of Circulating CD4^+^ T Cells during the Acute Phase of SIV Infection in Rhesus Macaques

**DOI:** 10.1371/journal.ppat.1002636

**Published:** 2012-04-12

**Authors:** Lia Vassena, Huiyi Miao, Raffaello Cimbro, Mauro S. Malnati, Giulia Cassina, Michael A. Proschan, Vanessa M. Hirsch, Bernard A. Lafont, Michel Morre, Anthony S. Fauci, Paolo Lusso

**Affiliations:** 1 Laboratory of Immunoregulation, National Institute of Allergy and Infectious Diseases, National Institutes of Health, Bethesda, Maryland, United States of America; 2 Human Virology Unit, DIBIT-HSR, Milano, Italy; 3 Biostatistics Research Branch, National Institute of Allergy and Infectious Diseases, National Institutes of Health, Bethesda, Maryland, United States of America; 4 Laboratory of Molecular Medicine, National Institute of Allergy and Infectious Diseases, National Institutes of Health, Bethesda, Maryland, United States of America; 5 Laboratory of Molecular Microbiology, National Institute of Allergy and Infectious Diseases, National Institutes of Health, Bethesda, Maryland, United States of America; 6 Cytheris, Issy-les Moulineaux, France; Emory University, United States of America

## Abstract

Although treatment with interleukin-7 (IL-7) was shown to transiently expand the naïve and memory T-cell pools in patients with chronic HIV-1 infection receiving antiretroviral therapy (ART), it is uncertain whether a full immunologic reconstitution can be achieved. Moreover, the effects of IL-7 have never been evaluated during acute HIV-1 (or SIV) infection, a critical phase of the disease in which the most dramatic depletion of CD4^+^ T cells is believed to occur. In the present study, recombinant, fully glycosylated simian IL-7 (50 µg/kg, s.c., once weekly for 7 weeks) was administered to 6 rhesus macaques throughout the acute phase of infection with a pathogenic SIV strain (mac251); 6 animals were infected at the same time and served as untreated controls. Treatment with IL-7 did not cause clinically detectable side effects and, despite the absence of concomitant ART, did not induce significant increases in the levels of SIV replication except at the earliest time point tested (day 4 post-infection). Strikingly, animals treated with IL-7 were protected from the dramatic decline of circulating naïve and memory CD4^+^ T cells that occurred in untreated animals. Treatment with IL-7 induced only transient T-cell proliferation, but it was associated with sustained increase in the expression of the anti-apoptotic protein Bcl-2 on both CD4^+^ and CD8^+^ T cells, persistent expansion of all circulating CD8^+^ T-cell subsets, and development of earlier and stronger SIV Tat-specific T-cell responses. However, the beneficial effects of IL-7 were not sustained after treatment interruption. These data demonstrate that IL-7 administration is effective in protecting the CD4^+^ T-cell pool during the acute phase of SIV infection in macaques, providing a rationale for the clinical evaluation of this cytokine in patients with acute HIV-1 infection.

## Author Summary

The development of highly effective cocktails of antiretroviral drugs has had a major impact on the survival and quality of life of individuals with HIV-1 infection. Yet, current protocols often fail to fully restore the immunologic function, a limitation that has prompted the clinical evaluation of immune-reconstitution agents, such as IL-7, as adjuvant therapies. To date, however, IL-7 has been tested exclusively in patients with chronic HIV-1 infection, while it appears that the immune system is irreparably damaged during acute primary infection, within the first few weeks after encountering the virus. We used a macaque model to show that treatment with IL-7 has beneficial effects if implemented during the acute phase of infection with SIV, the simian AIDS virus. Early administration of IL-7 was safe and effectively protected CD4^+^ T cells, the primary target cells for the virus, from the marked decline that typically occurs during acute SIV infection. Furthermore, IL-7 boosted the development of antiviral immune responses. Thus, IL-7 might be an effective adjuvant therapy in acute HIV-1 infection, which can protect the pool of CD4^+^ T cells before it is irreversibly compromised by the action of the virus.

## Introduction

Although HIV-1 establishes a chronic active infection that evolves toward clinical immunodeficiency over a span of several years, accumulating evidence indicates that critical pathogenic events take place during the acute phase of infection, leading to a massive and seemingly irreversible depletion of CD4^+^ T cells, predominantly of the memory phenotype [1], [2]. A large fraction of the T-cell pool in the body is harbored in the gut-associated lymphoid tissue (GALT) [3], which has been identified as a primary anatomical site for CD4^+^ T-cell depletion in both HIV-1-infected patients [4]–[6] and SIV-infected nonhuman primates [1], [2], [7], [8]; yet, the loss of CD4^+^ T cells within the early phase of infection appears to be a systemic phenomenon that involves all secondary lymphoid organs [1], [9], [10]. Taken together, these observations suggest that interventions aimed at preventing or reducing the immunologic damage caused by HIV-1 would be most effective if implemented during the earliest stages of infection, before the pool of memory CD4^+^ T cells becomes irreversibly compromised.

In spite of extensive research over the past three decades, the mechanism of CD4^+^ T-cell depletion during the course of HIV-1 infection is still debated. Studies in SIV-infected macaques have highlighted the role of direct cytopathic effects of the virus during the course of acute primary infection [1], [9], [10]. However, indirect mechanisms, including bystander apoptosis, may also be important, as suggested by the increased levels of apoptosis detected in blood and lymphoid organs of macaques acutely infected with pathogenic SIV strains [2], [11]–[15], as well as in *ex vivo*-cultured T cells from individuals with acute HIV-1 infection [16]–[18]. Thus, the use of anti-apoptotic agents during primary HIV-1 infection may have beneficial effects for preserving the integrity of the CD4^+^ T-cell pool. We previously demonstrated that interleukin-7 (IL-7), a nonredundant cytokine that plays a critical role in the development and homeostasis of the T-lymphoid compartment of the immune system [19]–[21], effectively reduces the levels of spontaneous apoptosis in both CD4^+^ and CD8^+^ T cells from HIV-1-infected individuals [22]. In lymphopenic hosts, the levels of endogenous IL-7 increase, causing transient proliferation of naïve and central memory (CM) CD4^+^ and CD8^+^ T cells, which eventually leads to the reconstitution of the physiological T-lymphocyte pool [20]–[22]. Owing to these unique biological properties, IL-7 is currently under clinical investigation as an immune-reconstitution agent in various forms of natural and iatrogenic immunodeficiencies, including those associated with AIDS and cancer [23], [24]. Pre-clinical studies in macaques chronically infected with SIV [25]–[28], as well as clinical studies in patients with HIV-1 infection or receiving treatment with immunosuppressive antineoplastic drugs [29]–[32], have documented beneficial effects of short-term courses of IL-7 therapy, resulting in the proliferation and numerical expansion of naïve and CM CD4^+^ and CD8^+^ T cells in peripheral blood and secondary lymphoid organs. Whether adjuvant therapy with IL-7 may effectively lead to the long-term reconstitution of the immunologic function remains unclear. Moreover, IL-7 treatment has never been evaluated in acute HIV-1/SIV infection, a phase in which it may still be possible to avert the seemingly irreversible immunologic damage caused by massive viral replication prior to the appearance of virus-specific adaptive immune responses.

In the present study, we administered fully glycosylated simian IL-7 to rhesus macaques during the acute phase of infection with a pathogenic SIV strain (mac251). The concomitant use of ART was deliberately avoided in order to exclude its confounding effects on pathogenesis since ART would have suppressed SIV replication, thereby preventing the pathologic depletion of CD4^+^ T-cells and making it impossible to evaluate the CD4-protective effects of IL-7. Another important goal of our study was to examine the effects of IL-7 on SIV replication since IL-15, a related common-γ-chain cytokine, was recently shown to dramatically increase SIV replication and accelerate disease progression when administered to acutely-infected macaques [33]. Our results demonstrate that treatment with IL-7 during the acute phase of SIV infection is safe and effective in preventing the decline of circulating naïve and memory CD4^+^ T cells without causing major increases in the levels of SIV replication.

## Results

### Safety and pharmacokinetics of glycosylated macaque IL-7

**Figure 1:**
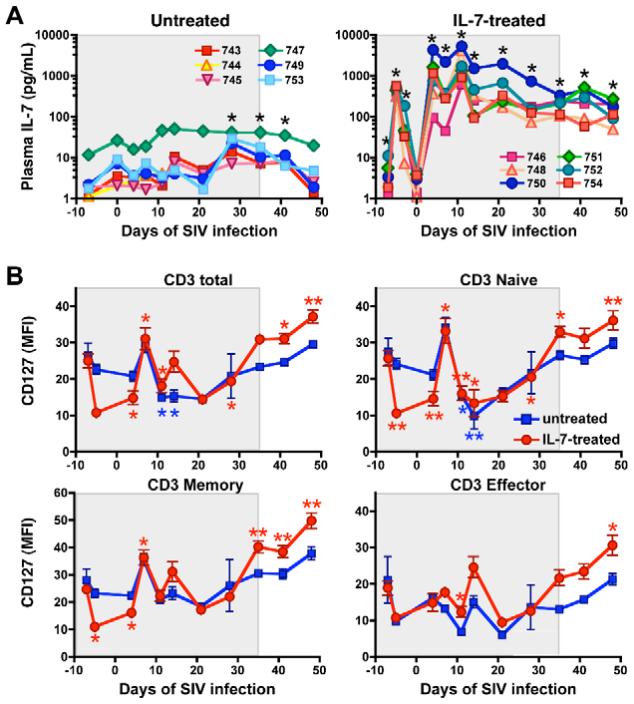
IL-7 pharmacokinetics and CD127 expression in circulating T cells in IL-7-treated and untreated animals. (A) Plasma IL-7 concentrations in IL-7-treated and untreated macaques. IL-7-treated animals received 7 weekly injections of 50 µg/kg of body weight of a recombinant, fully glycosylated form of simian IL-7 (the grey shaded area indicates the IL-7-treatment period). The asterisks denote significant differences with baseline values (p<0.05 by paired Student's t test). (B) CD127 expression on total, naïve, memory and effector CD3^+^ T cells from untreated (blue) and IL-7-treated (red) animals. Average values of mean fluorescence intensity (MFI) ± standard error of the mean (SEM) from each group of macaques are shown. Blue and red asterisks denote significant differences with baseline values (day −7) in untreated and treated animals, respectively (* p<0.05, ** p<0.01, by paired Student's t test).

None of the 6 rhesus macaques treated with IL-7 exhibited adverse clinical side effects throughout the treatment period. After the first IL-7 injection (day −7 relative to SIV infection), plasma IL-7 levels peaked on day −5 to return to baseline on day 0 (Figure 1A). The two subsequent injections (day 0 and day 7) induced higher peak levels of plasma IL-7 and a greater area under the curve (AUC), resulting in markedly elevated trough levels before each of the following injections. This pattern likely reflects the initial distribution of the cytokine to a high-affinity compartment that was saturated upon subsequent injections. Increased plasma levels of IL-7, albeit considerably lower than in IL-7-treated animals, were also observed in untreated animals starting on day 28 post-infection in parallel with the most pronounced reductions in circulating lymphocyte counts (Figure 1A). No significant correlations were found between plasma levels of IL-7 and various immunological parameters, including circulating CD4^+^ and CD8^+^ T-cell counts and expression of the specific chain of the IL-7 receptor (IL-7Rα or CD127) (data not shown). In agreement with previous studies [25]–[32], IL-7 treatment initially caused a significant downmodulation of the IL-7 receptor (CD127) in both naïve and memory T cells (Figure 1B).

### IL-7 treatment during the acute phase of SIV infection did not cause major increases in the levels of SIV replication and proviral SIV DNA load

**Figure 2:**
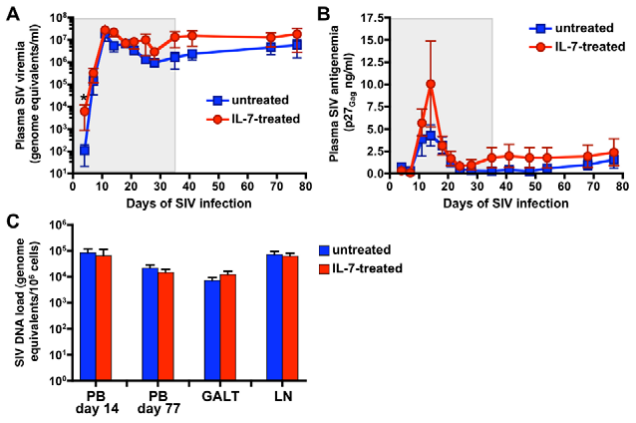
Effect of IL-7 treatment on SIV replication. Mean levels (± SEM) of SIV plasma viremia (A) and p27_Gag_ antigenemia (B) in untreated (blue) and IL-7-treated (red) animals. No significant differences were observed between the two groups of animals, with the only exception of SIV plasma viremia on day 4 post-infection, when IL-7-treated animals showed higher levels compared to untreated controls (indicated by the asterisk; p<0.05, by Wilcoxon rank sum test). The grey-shaded area indicates the IL-7-treatment period. (C) Mean number of genome equivalent copies (± SEM) of SIV proviral DNA in mononuclear cells from peripheral blood (days 14 and 77 post-infection) and lymphoid tissues (GALT, days 14–16; axillary lymph nodes, days 25–27 post-infection). No significant differences were observed between the two groups of animals, p>0.05 by Wilcoxon rank sum test.

Comparison of the two groups of animals showed that treatment with IL-7 did not induce significant increases in the levels of SIV plasma viremia at any time during the acute phase of infection and the follow-up period, including the peak of viral replication, the viral set point and the AUC, with the only exception of the earliest time point analyzed (day 4 post-infection; p = 0.043 for the comparison between the two groups of animals by Wilcoxon rank sum test) (Figure 2A). However, there was a trend towards higher levels of viremia in IL-7-treated animals, particularly on days 35 and 41 post-infection, even though the statistical p values remained far below the threshold for significance (Table S1 in Text S1). Likewise, the two groups of animals showed no significant differences in the levels of SIV p27 antigenemia (Figure 2B and Table S1 in Text S1) and proviral SIV DNA load detected in blood mononuclear cells on days 14 and 77, in the GALT (ileum) on days 14–16, and in axillary lymph nodes on days 25–27 post-infection (Figure 2C and Table S1 in Text S1). All the animals developed SIV-specific IgG antibodies, which became detectable between day 11 and day 21 of infection (data not shown).

During the follow-up period, 4 animals (two untreated and two IL-7-treated) developed early signs of progression to AIDS (rapid progressors [RP]) and were euthanized for terminal disease within 5 months of infection. The RP course has been suggested to represent a unique form of SIV disease, distinct from that of conventional progressors (CP) and associated with an unusual pathogenesis characterized by higher levels of SIV viremia, massive SIV replication in mononuclear phagocytic cells rather than in CD4^+^ T cells (with consequent lack of depletion of CD4^+^ T cells), and severe SIV-related enteropathy in the absence of opportunistic infections [34]. Additional data on RP animals are presented in Supplementary Data and Figure S1 in Text S1. Since the presence of RP animals could be a confounding factor in our study, all the virological and immunological data were analyzed both with the inclusion and after the exclusion of the 4 RP animals. When the analysis of SIV plasma viremia and antigenemia was restricted to CP animals, the statistical comparisons between IL-7-treated and untreated animals did not show any significant changes from those obtained with the inclusion of all the animals (Figure S2A in Text S1).

### IL-7 treatment during the acute phase of SIV infection prevented the decline of circulating naïve and memory CD4^+^ T cells

**Figure 3:**
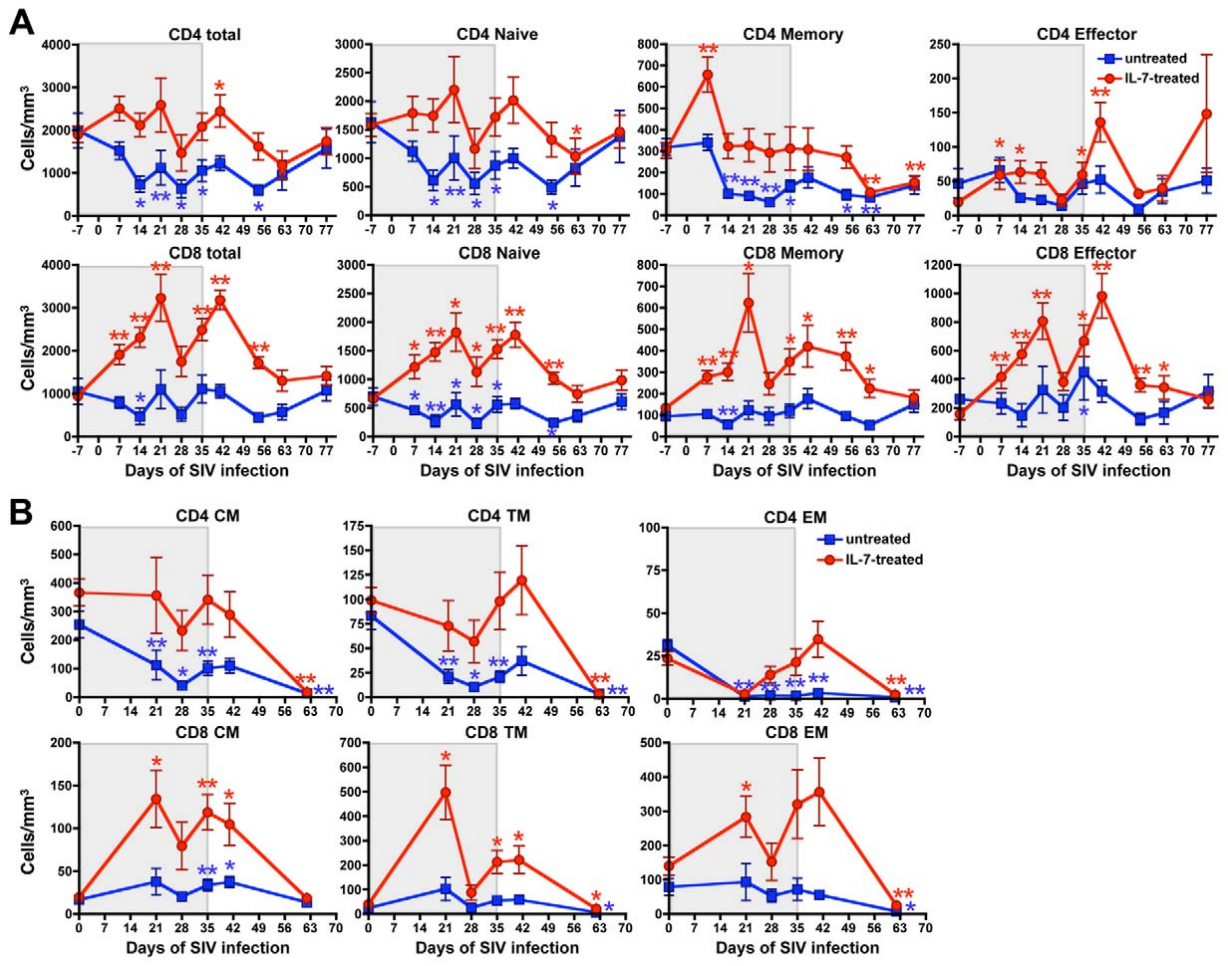
Effect of IL-7-treatment on peripheral blood T-lymphocyte kinetics. (A) Mean absolute numbers (± SEM) of circulating total, naïve, memory and effector CD4^+^ and CD8^+^ T cells in untreated (blue) and IL-7-treated (red) animals. The naïve (CD28^+^95^−^), memory (CD28^+^95^+^) and effector (CD28^−^95^+^) T-cell subsets were identified using a combination of mAbs against CD28 and CD95. (B) Subset analysis of memory CD4^+^ and CD8^+^ T cells in untreated (blue) and IL-7-treated (red) animals. The various memory T-cell subsets (central memory, CM; transitional memory, TM; and effector memory, EM) were identified using a combination of CD28, CD95, CD62L and CD197/CCR7. Absolute counts for each T-lymphocyte subpopulation were calculated by multiplying the percent values obtained by flow cytometry by the total lymphocyte counts obtained from the complete blood counts (CBC). The grey shaded area indicates the IL-7-treatment period; blue and red asterisks denote significant differences versus baseline values in untreated and IL-7-treated animals, respectively (* p<0.05, ** p<0.01, by paired Student's t test).

While all the animals in the untreated group experienced a marked and sustained decline of circulating CD4^+^ T lymphocytes starting at the time of peak SIV replication (day 14 post-infection), IL-7-treated animals showed no decline of CD4^+^ T-cell counts over the entire treatment period, with even a significant increase, relative to baseline, on day 41 (Figure 3A). When the two groups of animals were compared, IL-7-treated macaques had significantly higher absolute numbers of peripheral CD4^+^ T cells at several time points, including day 14 post-infection (statistical data not shown); similar results were obtained by comparison of the changes in CD4^+^ T-cell counts from baseline in IL-7-treated versus untreated animals (Figure S3 in Text S1).

To better characterize the effects of IL-7 on CD4^+^ T cells, the naïve, memory and effector subpopulations were analyzed separately. In the absence of IL-7 treatment, SIV infection caused significant decreases in the absolute numbers of naïve and memory CD4^+^ T cells, compared to pre-infection levels, throughout the acute phase of the infection (Figure 3A). In contrast, IL-7 caused an initial increase in memory CD4^+^ T cells (day 7) and subsequently prevented the decline of both naïve and memory CD4^+^ T cells throughout the treatment period; effector CD4^+^ T cells were increased at several time points (Figure 3A). However, the protective effects of IL-7 were not sustained after treatment interruption with both naïve and memory CD4^+^ T cells becoming significantly decreased, compared to baseline values, on day 62 post-infection, 4 weeks after the last injection of IL-7 (Figure 3A). When the absolute numbers of circulating naïve, memory and effector CD4^+^ T cells in the two groups of animals were compared, significant differences were detected at several time points (statistical data not shown); similar results were obtained by comparison of changes from baseline in IL-7-treated versus untreated animals (Figure S3 in Text S1). The statistical differences between treated and untreated animals remained significant when the analysis of total, naïve, memory and effector CD4^+^ T cells was restricted to CP animals, after exclusion of the 4 RP animals (Figure S2B in Text S1).

A detailed subset analysis of the memory CD4^+^ T-cell population was performed at selected time points. While in untreated animals all memory CD4^+^ T-cell subsets (CM, transitional memory [TM] and effector memory [EM]) dramatically declined during the acute phase of infection, none of these subsets was quantitatively reduced in animals receiving IL-7 (Figure 3B). The protective effects of IL-7 on memory CD4^+^ T cells were not sustained after treatment interruption as shown by the significant decline of all three subpopulations, compared to baseline, on day 62 post-infection (Figure 3B).

### IL-7 treatment expanded all subsets of circulating CD8^+^ T cells

IL-7-treated animals experienced sustained increases in all subsets of circulating CD8^+^ T cells throughout the acute phase of infection, whereas a transient decline of naïve and memory CD8^+^ T cells was observed in untreated animals (Figure 3A). When the two groups of animals were compared, IL-7-treated monkeys showed higher absolute numbers of peripheral CD8^+^ T cells at several time points (statistical data not shown); similar results were obtained by comparison of changes from baseline between IL-7-treated and untreated animals (Figure S3 in Text S1). Subset analysis of memory CD8^+^ T cells revealed no major changes in untreated animals, whereas IL-7 induced significant increases in all memory CD8^+^ T-cell subsets (Figure 3B). However, on day 62 post-infection, CM CD8^+^ T cells returned to baseline values, while TM and EM CD8^+^ T cells were significantly decreased in both IL-7-treated and untreated animals (Figure 3A and 3B). The statistical differences between treated and untreated animals remained significant when the analysis of total, naïve, memory and effector CD8^+^ T cells was restricted to CP animals, after exclusion of the 4 RP animals (Figure S2B in Text S1).

### Repeated IL-7 administrations induced only a transient increase in T-cell proliferation but persistent signs of apoptosis reduction

**Figure 4:**
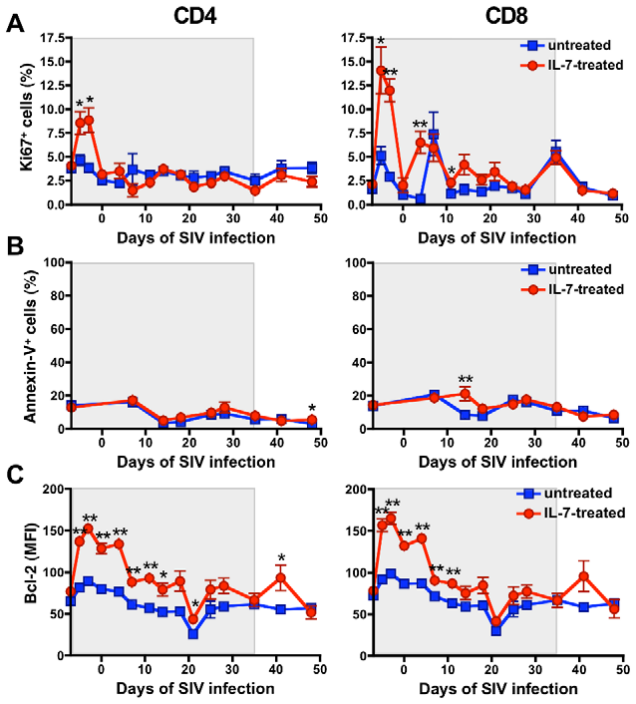
Effect of IL-7-treatment on cellular proliferation, apoptosis and Bcl-2 expression in peripheral blood T cells. (A) Mean levels (± SEM) of cellular proliferation, as measured by expression of the cell-cycling marker Ki67, in CD4^+^ and CD8^+^ T cells freshly isolated from untreated (blue) and IL-7-treated (red) animals. (B) Mean levels (± SEM) of spontaneous apoptosis, as measured by Annexin-V binding, in circulating CD4^+^ and CD8^+^ T cells from untreated (blue) and IL-7-treated (red) animals. (C) Average MFI levels (± SEM) of Bcl-2 expression in circulating CD4^+^ and CD8^+^ T cells from untreated (blue) and IL-7-treated (red) animals. The grey shaded area indicates the IL-7-treatment period. The asterisks denote significant differences between untreated and IL-7-treated animals (* p<0.05, ** p<0.01, by Wilcoxon rank sum test).

Longitudinal analysis of the levels of proliferation in freshly isolated peripheral blood T cells revealed that IL-7 treatment induced only a transient increase in the proportion of Ki67-expressing CD4^+^ T cells during the first week of treatment (day −5 and −3 prior to SIV infection), which returned to baseline levels at the time of SIV inoculation (day 0); remarkably, there was no further increase in proliferation after any of the subsequent IL-7 injections (Figure 4A). In contrast, CD8^+^ T cells showed elevated Ki67 expression both before SIV inoculation and at two time points (day 4 and 11) after infection, even though the proportion of cycling cells returned to baseline thereafter (Figure 4A).

Analysis of apoptosis in circulating T cells by Annexin-V binding did not show significant elevations, compared to baseline, in either untreated or IL-7-treated animals throughout the acute phase of SIV infection (Figure 4B), suggesting that the peripheral blood compartment is largely spared from *in vivo* apoptosis during primary infection; however, IL-7-treated animals exhibited significant and sustained increases in the intracellular levels of the anti-apoptotic protein Bcl-2 in both CD4^+^ and CD8^+^ T cells during the first three weeks of treatment (Figure 4C). Representative histograms illustrating Ki67 expression, Annexin-V binding and Bcl-2 expression in CD4^+^ and CD8^+^ T cells from one untreated (#749) and one IL-7-treated (#746) animals are shown in Figure S4 in Text S1. Altogether, these results suggested that numerical expansion due to proliferation was not a major factor contributing to the lack of decline of circulating CD4^+^ T cells seen in IL-7-treated animals while a decreased sensitivity to apoptosis was likely involved. In contrast, both proliferation and apoptosis reduction may have contributed to the sustained numerical increases documented in circulating CD8^+^ T cells.

### Effects of IL-7 treatment in lymphoid tissues

**Figure 5:**
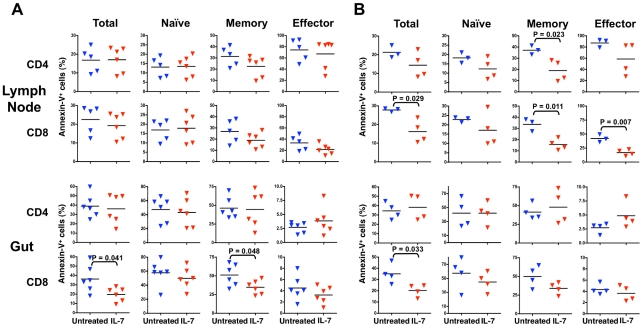
Frequency of CD4^+^ and CD8^+^ T-cell subsets and analysis of spontaneous apoptosis in terminal ileum and lymph node biopsies from untreated and IL-7-treated macaques. Lymph node (upper panels) and gut (lower panels) biopsies were obtained from all animals on days 25–27 and on days 14–16 post-infection, respectively. (A) Mean levels of spontaneous apoptosis, as measured by Annexin-V binding, in total, naïve, memory and effector CD4^+^ and CD8^+^ T cells isolated from axillary lymph node and terminal ileum biopsies from untreated (blue) and IL-7-treated (red) animals. The differences between untreated and IL-7-treated animals were analyzed by unpaired t-test (similar results were obtained by Wilcoxon rank sum test). (B) Mean levels of spontaneous apoptosis, as measured by Annexin-V binding, on total, naïve, memory and effector CD4^+^ and CD8^+^ T cells freshly isolated from axillary lymph node and terminal ileum biopsies from untreated (blue) and IL-7-treated (red) animals, after the exclusion of 4 rapid progressor (RP) animals. The differences between untreated and IL-7-treated animals were analyzed by unpaired t-test.

Next, we studied the effects of IL-7 in peripheral lymphoid tissues. Lymph node biopsies were collected from treated and untreated macaques on days 25–27 post-infection. The relative proportions of total, naïve, memory and effector CD4^+^ and CD8^+^ T cells in lymph nodes were not significantly different between IL-7-treated and untreated macaques, and the level of Ki67 expression was very low (<1%) in both groups of animals (data not shown). Analysis of T-cell apoptosis by Annexin-V binding and intracellular Bcl-2 expression did not show significant differences between the two groups of animals despite a trend toward reduced Annexin-V binding in memory CD4^+^ and CD8^+^ T cells and effector CD8^+^ T cells from IL-7-treated macaques (Figure 5A, upper panel); however, these differences became statistically significant when the analysis was restricted to CP animals, after exclusion of the 4 RP animals (Figure 5B, upper panel). In accordance with the Annexin-V data, lymph node CD4^+^ T cells from IL-7-treated macaques showed higher levels of intracellular Bcl-2 when RP animals were excluded from the analysis (data not shown).

Terminal ileum biopsies were obtained from all animals on days 14–16 post-infection. The yield of CD3^+^ T cells from these biopsies was highly variable (range = 1.2–15.0% of the total cell populations), underscoring the inherent difficulties in obtaining mucosal specimens with comparable representation of the GALT via retrograde ileoscopy. Regardless of this limitation, no significant differences were detected in the relative proportions of total, naïve, memory and effector CD4^+^ T cells, as well as in the CD4/CD8 ratio in the intestinal tissues of IL-7-treated vs. untreated macaques (data not shown); likewise, the proportion of Annexin-V-positive CD4^+^ T cells was similar in the two groups (Figure 5A, lower panel). In contrast, the proportion of Annexin-V-positive CD8^+^ T cells was lower in IL-7-treated animals (p = 0.041), primarily due to a reduction of apoptosis among memory CD8^+^ T cells (p = 0.048) (Figure 5A, lower panel), associated with a lower proportion of apoptosis-prone naïve CD8^+^ T cells (p = 0.024) (data not shown). The difference in the proportion of Annexin-V-positive CD8^+^ T cells remained significant when RP animals were excluded from the analysis (Figure 5B, lower panel).

### IL-7 treatment elicited earlier and stronger SIV-specific CD4^+^ and CD8^+^ T-cell responses

**Figure 6:**
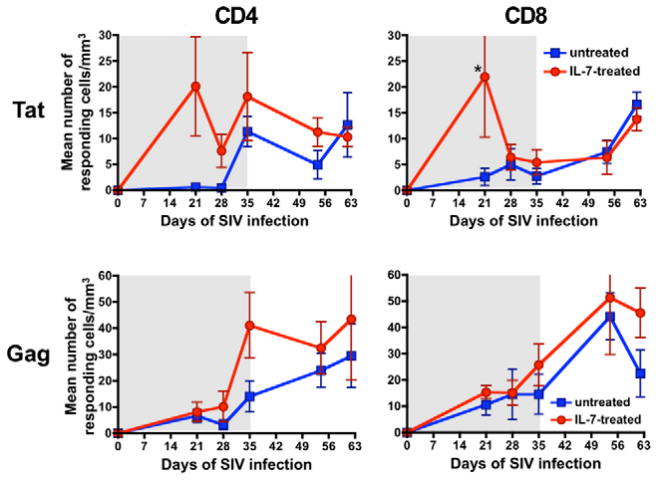
SIV-specific T-cell responses in untreated and IL-7-treated macaques. Mean absolute numbers (± SEM) of total CD4^+^ and CD8^+^ T cells responding to overlapping peptides derived from the Tat and Gag proteins of SIV in untreated (blue) and IL-7-treated (red) animals. The asterisk indicates a significant difference between total responses in IL-7-treated vs. untreated macaques.

SIV-specific T-cell responses were evaluated at multiple time points during and after the IL-7 treatment period by measuring the intracellular production of IFN-γ, IL-2 and MIP-1β in CD4^+^ and CD8^+^ T cells stimulated with peptide pools derived from the SIV Tat and Gag proteins. SIV-specific T-cell responses were detected at multiple time points in all animals, while we were unable to document the presence of SIV-neutralizing antibodies in serum at any time during acute primary infection in both untreated and IL-7-treated macaques (data not shown). The total number of Tat-specific CD8^+^ T cells at the first time point analyzed (day 21 post-infection) was significantly higher in IL-7-treated than in untreated animals (p = 0.017), whereas the difference in Tat-specific CD4^+^ T-cell responses was close to but did not reach statistical significance (p = 0.051) (Figure 6). Overall, IL-7-treated animals displayed higher numbers of Gag-responding CD4^+^ and CD8^+^ T cells at several time points, but the differences did not reach statistical significance (Figure 6).

**Figure 7:**
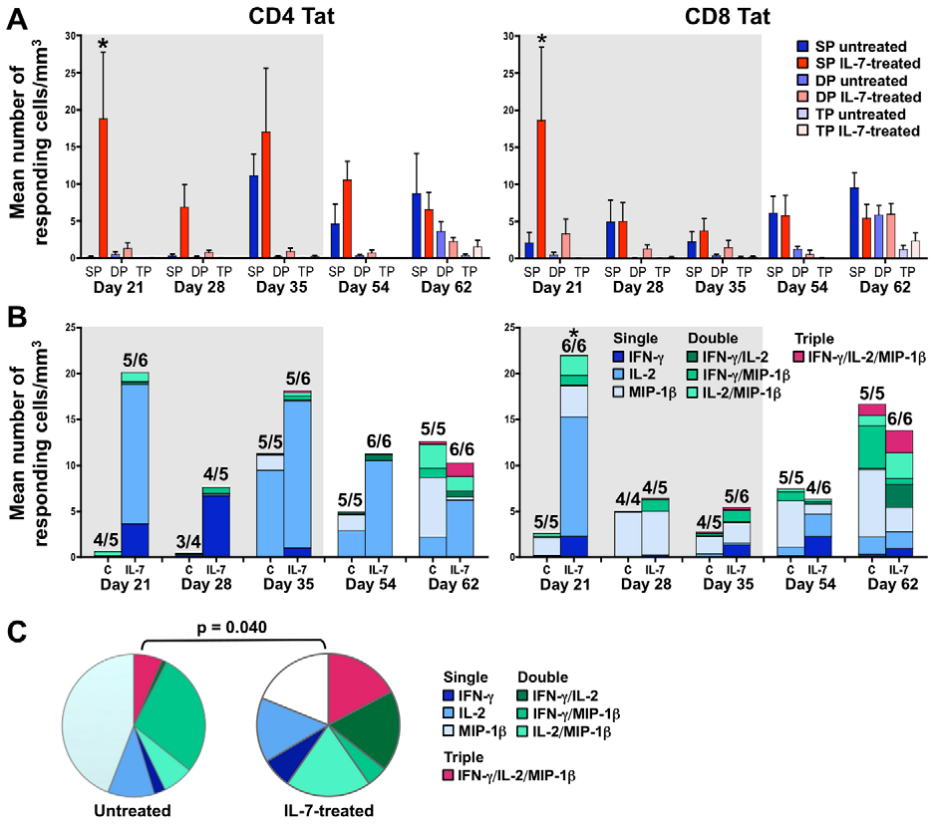
Qualitative analysis of SIV-specific T-cell responses in untreated and IL-7-treated macaques. (A) Mean absolute numbers (± SEM) of CD4^+^ and CD8^+^ T cells producing one cytokine (single-producer, SP), two cytokines (double-producer, DP) or all three cytokines (triple-producer, TP) in response to SIV Tat peptide stimulation in untreated (shades of blue) and IL-7-treated (shades of red) animals. The asterisks indicate significant differences between SP T cells in untreated vs. IL-7-treated animals, as analyzed by Wilcoxon rank sum test. (B) Mean absolute numbers of SIV Tat-responding CD4^+^ and CD8^+^ T cells in untreated (U) and IL-7-treated (IL-7) animals. The bars indicate the mean numbers of total responding cells; the colors indicate the mean numbers of IFN-γ, IL-2, or MIP-1β SP cells (shades of blue), IFN-γ/IL-2, IFN-γ/MIP-1β or IL-2/MIP-1β DP cells (shades of green), and IFN-γ/IL-2/MIP-1β TP cells (purple). The numbers above each bar indicate the fraction of monkeys that gave a measurable response over background to SIV Tat peptides at the corresponding time point. The grey shaded areas indicate the IL-7-treatment period. The asterisk indicates a significant difference between total responses in IL-7-treated vs, untreated macaques. (C) Qualitative analysis of SIV Tat-specific CD8^+^ T-cell responses in IL-7-treated and untreated macaques on day 62 post-infection. The pie charts indicate the average contribution of the various functional subpopulations of Tat-responding CD8^+^ T cells (single-, double- and triple-producing, SP, DP, TP, respectively) to the total number of Tat-responding cells in untreated and IL-7-treated animals at day 62 post-infection. The p value was calculated by permutation analysis using the Spice software.

Qualitative analysis of SIV-specific T-cell responses revealed that initially most Tat-specific (Figure 7A) and Gag-specific (Figure S5 in Text S1) CD4^+^ and CD8^+^ T cells were monofunctional in both groups of animals, producing a single cytokine (single-producer, SP). When SP cells were analyzed separately from double- and triple-producer cells (DP and TP), the difference between IL-7-treated and untreated animals on day 21 was significant for both CD4^+^ and CD8^+^ Tat-specific T cells (p = 0.030 and 0.017, respectively; Figure 7B). The quality of the T-cell responses evolved over time, with both Tat-specific (Figure 7B) and Gag-specific (Figure S5 in Text S1) T cells acquiring some degree of polyfunctionality over time. This phenomenon was more prominent among IL-7-treated animals, as indicated by a significant difference in the proportion of the various functional subpopulations of Tat-specific CD8^+^ T cells on day 62 post-infection (Figure 7C).

## Discussion

While the progressive refinement of multi-drug ART protocols has led to extraordinary advances in the treatment of chronic HIV-1 infection, there is still uncertainty as to whether a complete reconstitution of the immunologic function can be achieved even after years of sustained virologic suppression [35]. The often incomplete immunologic reconstitution in patients treated with ART, which is linked to an increased incidence of adverse clinical events [36], has prompted consideration of adjuvant therapies, including treatment with cytokines of the common-γ-chain family, such as IL-2, IL-7 and IL-15, which are known to induce T-cell expansions. However, the ability of such treatments to regenerate fully competent naïve and memory T-cell pools remains questionable as multiple lines of evidence indicate that the initial damage caused by HIV-1 during acute primary infection is irreversible [1], [2], [10], marking a critical event in the pathogenesis of HIV-1 disease and precluding the success of any restoration attempts enacted during the chronic phase. These considerations underscore the need to aim adjuvant treatment strategies toward immunologic preservation rather than reconstitution and, therefore, to implement such strategies at the earliest possible time after the diagnosis of HIV-1 infection. In this study, we used a macaque model to demonstrate that treatment with IL-7, the principal T-cell homeostatic cytokine, can prevent the dramatic decline of circulating naïve and memory CD4^+^ T cells that occurs during acute primary SIV infection. Although in the clinical setting IL-7 would presumably be associated with ART, as it was done in phase-1 studies in patients with chronic HIV-1 infection [29], [30], we deliberately avoided the use of virus-suppressive drugs in order to allow for an unbiased evaluation of the effects of IL-7 on CD4^+^ T-cell depletion, which is the hallmark of HIV-1/SIV-induced pathogenesis during primary infection. In fact, ART by itself would have prevented the loss of CD4^+^ T cells, limiting the scope of our study. The fact that in acutely-infected macaques treatment with IL-7 was able to protect naïve and memory CD4^+^ T cells even in the absence of ART suggests that the combination of IL-7 with virus-suppressive drugs would be even more effective in maintaining the integrity of the CD4^+^ T-cell pool during one of the most critical phases in the pathogenesis of HIV-1 disease.

To elucidate the mechanisms responsible for the CD4-protective effects of IL-7, we examined the kinetics of CD4^+^ T-cell proliferation and apoptosis, as well as the induction of SIV-specific cellular immune responses during the course of IL-7 treatment. Some levels of proliferation, albeit low, were documented following the first injection of IL-7, presumably leading to the observed initial expansion of the memory CD4^+^ T-cell subset. However, in agreement with data of IL-7 treatment in chronically SIV-infected macaques [28], no further proliferation was seen after any subsequent IL-7 injection, suggesting that repeated administrations of fully glycosylated IL-7 at weekly intervals may in fact induce tachyphylaxis, at least concerning the proliferative effects of the cytokine. Conversely and in agreement with our previous *ex vivo* findings [22], several observations pointed to a reduction of apoptosis as one of the mechanisms responsible for CD4^+^ T-cell preservation in our IL-7-treated macaques. First, while the levels of apoptosis in peripheral blood T cells did not change significantly during primary SIV infection in either treated or untreated animals, preventing the evaluation of the anti-apoptotic effects of IL-7 in this compartment, there was a sustained increase in the expression of the anti-apoptotic protein Bcl-2, a marker of increased resistance to apoptosis, in circulating CD4^+^ and CD8^+^ T cells from IL-7-treated animals. Second, analysis of secondary lymphoid tissues (lymph nodes and GALT) demonstrated a reduction of apoptosis in selected CD4^+^ and CD8^+^ T-cell populations in IL-7-treated macaques, even though these tissues were only sampled at a single time point during the acute phase due to the inherent difficulties in obtaining multiple tissues from the same animal within a short period of time. This limitation reduced our chances to document the full spectrum of anti-apoptotic effects of IL-7. Of note, analysis of the GALT at the time of peak SIV replication demonstrated that IL-7 reduced the proportion of Annexin-V-binding CD8^+^ T cells, particularly of the memory phenotype, but not CD4^+^ T cells. An important caveat that must be considered in this setting is the inconsistent quality of ileum specimens obtained by retrograde ileoscopy, which have a high probability of sampling error due to a markedly variable yield of lymphoid cells. Regardless, the fact that we did not observe even a trend toward reduced apoptosis among GALT CD4^+^ T cells may reflect the fact that IL-7 is inactive against the direct cytopathic effect of the virus, which has been identified as the principal mechanism responsible for CD4^+^ T-cell depletion in the gut during the acute phase of the infection, whereas the role of apoptosis in this context remains controversial [1], [2]. Although a more extensive investigation is warranted to confirm this hypothesis, our data suggest that IL-7 treatment prevented the decline of circulating CD4^+^ T cells during the acute phase of SIV infection by inducing an initial expansion of these cells combined with their subsequent preservation via sustained pro-survival effects.

Since IL-7 seemed to effectively preserve the pool of CD4^+^ T cells, which are the main cellular targets for SIV, one could have anticipated considerable increases in the levels of virus replication in acutely infected macaques treated with IL-7. However, despite the absence of concomitant ART, we did not observe dramatic increases in the levels of SIV replication during both the treatment phase and the follow up period, with the only exception of the earliest time point analyzed (day 4 post-infection) in which the levels of SIV plasma viremia were significantly higher in IL-7-treated than in untreated macaques. A trend toward higher levels of SIV plasma viremia was seen at certain time points, particularly day 35 and day 41 post-infection, but it did not reach statistical significance even after exclusion from the analysis of the 4 animals with rapid disease progression. We cannot rule out that this lack of statistical significance was due to the low number of animals included in the study (n = 6 per group), even though the p values remained far below the threshold for significance. Nevertheless, it is undisputable that, if an enhancing effect of IL-7 on SIV replication did occur, this effect was far less dramatic than that documented for a related common-γ-chain cytokine, IL-15, in a similar experimental setting [33]. Indeed, IL-15 treatment of macaques during the acute phase of SIV infection was shown to induce a ∼3 log increase in viral load, most likely due to increased CD4^+^ T-cell proliferation and activation, and to accelerate the disease progression [33]. Various hypotheses can be formulated to explain these striking differences between the outcomes of IL-7 and IL-15 treatment. First, the lack of strong induction of CD4^+^ T-cell proliferation by IL-7, unlike IL-15, which was detectable only transiently and only after the first IL-7 injection; of note, this transient proliferative effect of IL-7 could have been responsible for the significant increase in SIV plasma viremia at the earliest time point tested (day 4 post-infection). Second, the induction of early and vigorous SIV Tat-specific CD4^+^ and CD8^+^ T-cell responses in IL-7-treated animals, as compared to untreated animals, associated with the proliferation, expansion and activation of all CD8^+^ T-cell subpopulations. Since Tat is a regulatory protein expressed during the early phase of the viral life cycle, Tat-specific T-cell responses presumably were able to halt the infection before completion of a full replicative cycle, thereby preventing the spread of infectious virus to other target cells. Of note, a pronounced numerical increase was detected in EM CD8^+^ T cells, a functionally competent subset that was recently associated with effective vaccine-elicited protection in macaques [37]. Thus, it is plausible that the increased availability of target CD4^+^ T cells in IL-7-treated animals was counterbalanced by the effectiveness of virus-specific cell-mediated immune responses resulting in the observed minimal increases in SIV replication levels. An interesting correlate of our findings was provided by a recent study in mice acutely infected with LCMV, in which early treatment with IL-7 augmented the number and functionality of specific antiviral effector T cells, thereby reducing organ pathology and promoting viral clearance [38]. Conversely, it is also possible that the earlier and stronger Tat-specific CD4^+^ and CD8^+^ T-cell responses observed in IL-7-treated animals resulted from a more robust antigenic stimulation due to the higher amount of virus detected in these animals at day 4 post-infection; indeed, strong CD8^+^ T-cell responses were also detected in IL-15-treated animals, although they were incapable of controlling viral replication [33]. Thus, the different mechanisms of action of IL-7 and IL-15 on CD4^+^ T cells, with IL-15 inducing high levels of activation and proliferation mainly in memory CD4^+^ T cells, the preferential target of the virus, and IL-7 stimulating the proliferation and renewal of naïve T cells too, thereby contributing to the preservation and replenishment of the peripheral T-cell pool, may account for the divergent effects of treatment with these two cytokines. Although a more extensive investigation is warranted in the perspective of a potential clinical use of IL-7 in acute HIV-1 infection, it is important to emphasize that in a therapeutic regimen IL-7 would always be administered in combination with ART, thereby minimizing its possible enhancing effects on viral replication.

Our results may have implications for devising new treatment strategies for acute HIV-1 infection, whose clinical management remains a challenge. A critical hurdle is the inherent difficulty in identifying and treating patients at the earliest possible stage in order to reduce the massive HIV-1 replication that occurs before the development of virus-specific adaptive immune responses and its deleterious effects on the immune system. The extent of viral replication during the acute phase of SIV infection in macaques has indeed been identified as a critical determinant of the natural history of the disease [39]. Although early ART treatment was reported to be beneficial on the induction and maintenance of HIV-specific cellular immune responses [39]–[41], additional studies in patients [10], [42] and macaques [43] have shown limited effects of ART alone on T-cell preservation in the intestinal lamina propria, underscoring the importance of devising effective adjuvant therapies. Indeed, even if ART is promptly initiated during primary infection, its effects may not be sufficient for fully preventing the immunologic damage caused by HIV-1, particularly in the gastrointestinal tract, due to dishomogeneous drug biodistribution or inactivation by P-glycoproteins within the intestinal mucosa. Moreover, complete suppression of viral replication could take several weeks, and indirect mechanisms of cell destruction, such as bystander apoptosis, may remain active for some time after the virus has ceased to replicate. Our data provide a scientific basis for the clinical evaluation of IL-7, in combination with ART, for the treatment of acute primary HIV-1 infection.

## Materials and Methods

### Ethics statement

The animals were housed and fed according to regulations established in the Guide for the Care and Use of Laboratory Animals and the Animal Welfare Act. The animal experiments performed in this study were approved by the NIH Animal Care and Use Committee (ACUC).

### Animals and study design

Twelve colony-bred juvenile Rhesus macaques, housed at Bioqual Inc. (Rockville, MD), were divided in two groups, according to HLA haplotype and baseline CD4^+^ T-cell counts (Table S2 in Text S1): untreated controls (group 1) and IL-7-treated (group 2). Blood samples were obtained three times during the first week of IL-7 treatment, then twice weekly for the entire treatment period and then once per week; terminal ileum biopsies were obtained on day 14–16 post-infection; lymph node biopsies were obtained on day 25–27 post-SIV infection. Since one animal in the control group (H744) was lost on day 14 post-infection during intestinal biopsy, all the data from day 14 onwards refer to a total of 11 animals.

### IL-7 treatment and SIV infection

Recombinant glycosylated simian IL-7 (Cytheris, France) was employed in this study since it has a considerably longer half-life and greater stability (M. Morre et al., unpublished results) than the non-glycosylated form that had been employed in several previous studies [27], [28]. IL-7 was administered subcutaneously to the 6 monkeys included in group 2 at a concentration of 50 µg/kg of body weight once per week for a total of 7 consecutive administrations. To allow for the achievement of steady-state plasma levels of the cytokine prior to SIV infection, treatment was initiated 1 week before SIV inoculation (day −7). On day 0, all 12 monkeys were inoculated intravenously with 100 macaque infectious doses of the pathogenic strain SIV_mac251_, kindly provided by Dr. R. C. Desrosiers. Multiple clinical, immunological and virological parameters were monitored throughout the acute phase of infection, as well as for a follow-up period of 6 months post-infection.

### Measurement of plasma IL-7 concentrations

The concentration of IL-7 in serial plasma samples was measured using a high-sensitivity commercial ELISA (Quantikine HS, R&D Systems, Minneapolis, MN).

### Peripheral blood cell counts and flow cytometric analyses

Peripheral blood was collected under sterile conditions in vacutainer tubes with EDTA as anticoagulant and complete blood cell count with differential was performed by a commercial laboratory (Antech Diagnostics, Rockville, MD). Plasma was separated by spinning whole blood at 500×g for 20 min at 4°C without brake and stored at −80°C. PBMC were isolated by gradient centrifugation using Lymphocyte Separation Medium (LSM; MP Biomedicals). Blood was diluted in Phosphate Buffered Saline (PBS), stratified over LSM and centrifuged at 500×g for 25 min at 4°C without brake. The mononuclear cell ring was collected, and the PBMC were washed twice with PBS, counted and used for cytofluorimetric analyses. The following monoclonal antibodies (mAbs) were used for surface staining: CD28-FITC (clone CD28.2), CD4-PerCpCy5.5 (clone L200), CD95-APC or -PE (clone DX2), CD8-PECy7 (clone SK1), CD3-APCCy7 (clone SP34-2), all from BD Biosciences. The naïve (CD28^+^CD95^−^), memory (CD28^+^CD95^+^) and effector (CD28^−^CD95^+^) T-cell subsets were identified as previously described [43]. At selected time points, a more detailed characterization of the memory T-cell compartment was performed by using a combination of the following mAbs: CD45RA-FITC (clone 5H9), CD28-PECy7, CCR7-APC (clone 3D12), CD3-V450, CD4-APCH7 (all from BD Biosciences), and CD8-eFLOUR 605NC (clone RPA-T8; eBioScience). As illustrated in Figure S6 in Text S1, central memory T cells (T_CM_) were defined as CD45RA^−^ CCR7^+^ CD28^+^, transitional memory T cells (T_TM_) as CD45RA^−^ CCR7^−^ CD28^+^, and effector memory T cells (T_EM_) as CD45RA^−^ CCR7^−^ CD28^−^. Additional mAbs were used to measure the expression of other cell-surface markers, including the IL-7 receptor-α/CD127-PE (clone hIL-7R-M21), CCR5-PE (clone 3A9), CXCR4-PE (clone 12G5) and the activation markers HLA-DR-PE (clone L243/G46-6) and CD25-PE (clone M-A251) (all from BD Biosciences). Stained cells were analyzed by flow cytometry using a BD FACSCanto collecting a minimum of 100,000 events per sample. Flow data were analyzed using the Flowjo software (Tree Star) or FCS Express (DeNovo Software).

Apoptosis was assessed by measuring the levels of Annexin-V binding, using Annexin V-APC conjugated (BD Biosciences), after surface staining, as previously reported [22]. Bcl-2 expression levels and cellular proliferation were assessed as follows: after surface staining, the cells were fixed and permeabilized using BD Cytofix/Cytoperm solution (BD Biosciences) and incubated with mAbs anti-Bcl-2-PE (clone Bcl-2/100) and anti-Ki67-PE (clone B56) (BD Biosciences). Annexin-V binding and Bcl-2 expression (Mean Fluorescence Intensity, MFI) were evaluated on various CD4^+^ and CD8^+^ T-cell subpopulation by multicolor flow cytometry using a BD FACSCanto, collecting a minimum of 100,000 events per sample, and flow data were analyzed with the Flowjo software (Tree Star) or FCS Express (DeNovo Software) applying a progressive gating strategy. Naïve memory and effector T-cell subsets were identified using a combination of anti-CD95 and anti-CD28 antibodies [44] on CD3^+^CD4^+^ and CD3^+^CD8^+^ gated cells, as described above for surface staining experiments.

### Measurement of SIV plasma viremia, proviral DNA and antigenemia

Plasma SIV RNA levels were measured using a quantitative real-time RT-PCR assay. Viral RNA was purified from 280 µl of cell-free plasma using the QIAamp Viral RNA kit (Qiagen, USA), and stored at −80°C. The number of SIV RNA genome equivalents was determined using a single-tube real-time RT-RCR assay based on the AgPath-ID One-Step RT-PCR Kit (Applied Biosystems, USA), under the following reaction conditions: 10 min at 45°C (RT), 10 min at 95°C and 40 cycles of 15 sec at 95°C and 45 sec at 60°C (PCR). Primers (300 nm) and probe (200 nm) were specifically designed within the SIV gag gene to amplify a fragment of 91 bp. Forward primer: 5′-GCAGAGGAGGAAATTACCCAGT-3′; reverse primer: 5′-ATTTTACCCAGGCATTTAATGTTC-3′ (used for the RT phase); TaqMan MGB probe, FAM-labelled: 5′-ACAAATAGGTGGTAACTATG-3′. The copy number was determined by interpolation on a standard curve of a DNA plasmid carrying a fragment of the SIV gag gene containing the RT-PCR amplicon (serial 10-fold dilutions from 10^0^ copies/reaction to 10^6^ copies/reaction). Forward cloning primer: 5′-GCAGAGACACCTAGTGATGGAAAC-3′; reverse cloning primer: 5′-TCTCCCACACAATTTAACATCTG-3′.

The SIV proviral DNA load was measured by real-time PCR using the same primers and probe as in the plasma viremia assay. Normalization for cell number was performed by quantification of a non-polymorphic single-copy gene, CCR5. Additional details about the method have been reported [45].

SIV p27_Gag_ antigenemia was measured by using the SIV Core Antigen Assay (Coulter Corporation, Miami, USA) according to the manufacturer's instructions. All samples were initially tested undiluted and retested at 1∶10 dilution if necessary. Plates were read using an end-point protocol with a microplate spectrophotometer (Bio-Rad Instruments).

### Measurement of anti-SIV antibodies

Anti-SIV monkey IgGs were quantified using a home-made ELISA. Briefly, plates were coated overnight at 4°C with 1 µg/ml of total SIV protein extract, obtained by lysing with 1% Triton x-100 for 1 hour at room temperature the SIV isolate SIV_mac251_/SupT1-CCR5 CL30 (from the AIDS & Cancer Virus Program, NCI, NIH, Frederick). The following day the plates were washed and blocked for 1 hour. Samples were then added to each well, initially undiluted and re-tested diluted 1∶5 or 1∶20, whenever needed. Plates were then incubated with biotinylated goat anti-human IgG (Sigma-Aldrich, #B1140) and HRP-conjugated streptavidin (R&D Systems), and read at a wavelength of 450 nm with a reference set at 570 nm on a microplate spectrophotometer (Bio-Rad Instruments). The presence of neutralizing anti-SIV antibodies in the sera of infected untreated and IL-7-treated animals was tested using an envelope-mediated fusion assay with PM1 cells chronically infected with SIV_mac251_ as effector cells and Hela (TZM-bl) cells expressing human CD4 and CCR5 as target cells.

### Terminal ileum and lymph node biopsies

Ileum biopsies were performed by retrograde ileoscopy on day 14–16 post-infection. At least 6–8 punch biopsies were obtained from the terminal ileum of each animal, immediately placed in cold RPMI medium and then processed within 3 hours of excision. During the procedure, one animal (H744) suffered an intestinal perforation and was lost. Ileum biopsies were digested in Iscove's media supplemented with 2 mg/ml Type II collagenase (Sigma-Aldrich) and 1 U/ml DNase I (Sigma-Aldrich) for 30 min at 37°C. After digestion, the samples were passed through a 70 µm strainer, and the suspended cells were washed twice with RPMI media supplemented with 10% heat-inactivated FBS.

Lymph node excisional biopsies were performed on day 25–27 post-infection on axillary lymph nodes from all animals. Lymph nodes were cut longitudinally in two halves, one of which was stored at −80°C. The remaining half was finely minced using sterile scalpels and mechanically smashed to release lymphoid cells into the media. Cells were then washed, passed through a 70 µm strainer and stained for surface and intracellular markers as described above.

### Analysis of SIV-specific T-cell responses

SIV-specific CD4^+^ and CD8^+^ T-cell responses were analyzed by measuring intracellular cytokine production after antigen stimulation. Frozen samples of PBMC were thawed in RPMI 10% FBS and rested at 37°C for at least 5 hrs. Cells were then plated at 5×10^5^ cells/well in a 96-well round-bottom plate in RPMI 10% FBS in the presence of purified anti-CD28 and anti-CD49d mAbs both at a final concentration of 1 ng/ml. Two pools of SIV-gag peptides and one pool of SIV-tat peptides (from NIBSC, EVA Centre for AIDS reagents) at a final concentration of 1 µg/ml (each peptide) and *Staphylococcus aureus* Enterotoxin B (SEB, Sigma) as positive control at a final concentration of 2 µg/ml were added to the samples in a total volume of 200 µl. A negative control with no stimulation was included. After 1 hr incubation at 37°C, 5% CO_2_, 1 µl of Brefeldin A (BD GolgiPlug, BD Biosciences) was added to each well, and the plates were incubated for an additional 11 hrs. At the end of the incubation period the cells were transferred to 96-well V-bottom plates and washed twice with PBS before surface intracellular staining. Cells were stained first with LIVE/DEAD Fixable Dead Cell Stain reagent (Invitrogen by Life Technologies) at a concentration of 1 µl/10^6^ cells for 20 min at 4°C and then with the mAbs to lineage antigens (CD3-V450 and CD4-APCH7, BD Biosciences), before fixation and permeabilization with BD Cytofix/Cytoperm Buffer (BD Biosciences). After washing with BD PermWash Buffer (BD Biosciences), the cells were incubated for 20 min at 4°C with anti-MIP-1β-PE (clone D21-1351), anti-IFN-γ-PECy7 (clone P2G10) and anti-IL-2-APC (clone MQ1-17H12; all from BD Biosciences), washed again and analyzed on a BD FACSCanto instrument. T-cell responses were analyzed using Flowjo (Tree Star) and Spice softwares.

### Statistical analysis

Statistical analysis was conducted using the softwares SAS (version 9.1 for Windows), S-Plus (version 6.2 for Windows), StatView (version 5.0.1 for Macintosh) and GraphPad Prism (version 4.0b for Macintosh). Paired Student's t-tests were used for the comparison between different time points within the same animal group (untreated or IL-7-treated). Non-parametric Wilcoxon rank sum tests were used to analyze differences between IL-7-treated and untreated animals. To compare untreated and IL-7-treated animals with respect to changes from baseline to multiple time points simultaneously the O'Brien test was used (for a more detailed description see Supporting Information online).

## Supporting Information

Text S1 Text S1 contains Supplementary Methods (study design; statistical analysis), Supplementary Data (features of animals with rapid disease progression; T-cell dynamics), Table S1 (p values for SIV viremia and antigenemia), Table S2 (MHC haplotype and baseline T-cell counts in study groups), Figure S1 (SIV replication in conventional vs. rapid progressors), Figure S2 (data reanalysis after exclusion of rapid progressors), Figure S3 (T-cell changes from baseline), Figure S4 (representative Ki67, Annexin-V and Bcl-2 profiles), Figure S5 (SIV Gag-specific T-cell responses), Figure S6 (gating strategy for naïve and memory T cells). Text S1 also contains references and explanatory legends to Tables and Figures.
(PDF)
