# Supplementary material for: Treatment with IL-7 Prevents the Decline of Circulating CD4+ T Cells during the Acute Phase of SIV Infection in Rhesus Macaques
Source: PLoS Pathog. 2012 Apr 12;8(4):e1002636. doi: 10.1371/journal.ppat.1002636 (PMC3325214; doi:10.1371/journal.ppat.1002636)
Supplement: Text S1 — contains Supplementary Methods (study design; statistical analysis), Supplementary Data (features of animals with rapid disease progression; T-cell dynamics), Table S1 (p values for SIV viremia and antigenemia), Table S2 (MHC haplotype and baseline T-cell counts in study groups), Figure S1 (SIV replication in conventional vs. rapid progressors), Figure S2 (data reanalysis after exclusion of rapid progressors), Figure S3 (T-cell changes from baseline), Figure S4 (representative Ki67, Annexin-V and Bcl-2 profiles), Figure S5 (SIV Gag-specific T-cell responses), Figure S6 (gating strategy for naïve and memory T cells). Text S1 also contains references and explanatory legends to Tables and Figures. (PDF) [file ppat.1002636.s001.pdf]

SUPPORTING INFORMATION FOR:

Treatment with IL-7 Prevents the Decline of Circulating CD4^{+} T Cells during the Acute Phase of SIV Infection in Rhesus Macaques

Lia Vassena,[1,2] Huiyi Miao,[1] Raffaello Cimbro,[1] Mauro S. Malnati,[2] Giulia Cassina,[2] Michael A. Proschan,[3] Vanessa M. Hirsch,[4] Bernard A. Lafont,[5] Michel Morre,[6] Anthony S. Fauci,[1] and Paolo Lusso[1]

[1]Laboratory of Immunoregulation, [3]Biostatistics Research Branch and [4]Laboratory of Molecular Medicine, [5]Laboratory of Molecular Microbiology, National Institute of Allergy and Infectious Diseases, National Institutes of Health, Bethesda, Maryland 20892; [2]Human Virology Unit, DIBIT-HSR, 20132 Milano, Italy; and [6]Cytheris, Issy-les Moulineaux 92370, France

This file contains:

- Supplementary Methods
- Supplementary Data
- Supplementary References
- Tables S1-S2
- Figures S1-S6

SUPPLEMENTARY METHODS

MHC screening and study groups

A critical issue that we considered in the study design was the assignment of the 12 animals to the two study groups (i.e., IL-7 treatment and control) in order to avoid potential biases in SIV-disease susceptibility. Since previous work identified MHC class-I alleles with disease/viral load-protective (Mamu-A01, Mamu-A08, Mamu-B08) or -enhancing (Mamu-B01) properties [1-4], we pre-screened the animals for defined Mamu haplotypes. A positive score (+1) was attributed to the presence of Mamu-A01, A08 or B08; a negative score (-1) to the presence of Mamu-B01 (Table S2). The final study groups were balanced considering both MHC scores and baseline counts of peripheral blood $CD3^+$ and $CD4^+$ T lymphocytes (Table S2).

Statistical analysis using the O'Brien test

To compare untreated and IL-7-treated animals with respect to changes from baseline to multiple time points simultaneously we used the O'Brien test, which is a natural extension of the Wilcoxon rank sum test to accommodate multiple time points per subject. The analysis was used to compare simultaneously the changes from baseline to each time point during the treatment period (day 7, 14, 21, 28, and 35). First, we ranked changes from baseline to each of the time points separately, and then for each animal we averaged the ranks across the time points. To assess statistical significance, a permutation test was used. Under the null hypothesis of no effect of IL-7 treatment, one should not be able to distinguish treated monkeys from controls; the control monkeys could have been any group of 5 selected from the 11 total (excluding monkey #744 for which several time points were missing). Considering that there are 462 ways to select 5 subjects from 11, for each of these relabelings, we computed the difference between the average for animals labeled as "IL-7-treated" and the average for animals labeled as "untreated". Afterwards, we computed the proportion of relabeled datasets that produced a mean difference at least as extreme as the actual trial data.

SUPPLEMENTARY DATA

Rapid disease progression in 4 SIV-infected macaques: re-analysis of virological and immunological data after exclusion of rapid progressors

During follow-up, two monkeys in each group (H745, H749 in the untreated group; H751, H752 in the IL-7-treated group) developed early signs of SIV-disease progression and were euthanized for humanitarian reasons after a mean of 143.5 ± 20.4 days (range, 115-163). No specific MHC allele was over- or under-represented among these 4 animals. Albeit infrequently, SIV-infected macaques have been shown to undergo a rapid disease course characterized by high-level virus replication in cells of the mononuclear phagocytic lineage rather than in $CD4^+$ T cells [5]. In agreement with these previous observations, our rapid progressors (RP) showed persistently higher levels of SIV plasma viremia (Figure S1A) and antigenemia (Figure S1B), compared to conventional progressors (CP), as well as overall higher numbers of circulating $CD4^+$ T cells, even though the difference reached statistical significance only on day 62 post-infection (Figure S1C). Although it is impossible, without extending the study to a larger group of animals, to establish if the RP course was affected by IL-7 treatment, the fact that two animals in each group progressed rapidly suggests that IL-7 did not influence this unique form of SIV-disease evolution.

Since the rapid disease course has been associated with an unusual pathogenesis [5], which is likely influenced by genetic factors and insensitive to IL-7 treatment, we reasoned that the inclusion of RP animals could be a confounding factor in our study, and therefore we re-analyzed all the immunological and virological data after exclusion of these 4 animals. This re-analysis showed no major changes in the statistical comparisons between treated and untreated animals regarding SIV viremia and antigenemia (Figure S2A), $CD4^+$, $CD8^+$, naïve, memory and effector T cells in peripheral blood (Figure S2B), ileum and lymph nodes (data not shown). However, the exclusion of RP animals had important effects on the analysis of T-cell apoptosis in lymph nodes, as described in the main text.

Changes in T-cell subpopulations in acutely SIV-infected macaques

The O'Brien permutation test was used to simultaneously compare the changes in naïve, memory and effector $CD3^+$, $CD4^+$ and $CD8^+$ T cells from baseline to all the time points during the treatment period (day 7-35 post-infection) in IL-7-treated versus untreated animals. As shown in Figure S3A, this analysis demonstrated that the changes in the total numbers of $CD3^+$, $CD4^+$ and $CD8^+$ T cells were significantly different in IL-7-treated and untreated macaques (Figure S3A). Likewise, significant differences between IL-7-treated and untreated animals were also observed for naïve, memory and effector $CD4^+$ and $CD8^+$ T cells, as well as for naïve and effector $CD3^+$ T cells, but not for memory $CD3^+$ T cells (Figure S3B). However, when the analysis of changes was restricted to day 14 post-infection, the differences between IL-7-treated and untreated animals were significant for all T-cell subpopulations (not shown).

SUPPLEMENTARY REFERENCES

1. Muhl T, Krawczak M, Ten Haaft P, Hunsmann G, Sauermann U (2002) MHC class I alleles influence set-point viral load and survival time in simian immunodeficiency virus-infected rhesus monkeys. J Immunol 169: 3438-3446.

2. Pal R, Venzon D, Letvin NL, Santra S, Montefiori DC, et al. (2002) ALVAC-SIV-gag-pol-env based vaccination and macaque major histocompatibility complex class I (A*01) delay simian immunodeficiency virus SIVmac-induced immunodeficiency. J Virol 76: 292-302.

3. Loffredo JT, Maxwell J, Qi Y, Glidden CE, Borchardt GJ, et al. (2007) Mamu-B*08-positive macaques control simian immunodeficiency virus replication. J Virol 81: 8827-8832.

4. Mothe BR, Weinfurter J, Wang C, Rehrauer W, Wilson N, et al. (2003) Expression of the major histocompatibility complex class I molecule Mamu-A*01 is associated with control of simian immunodeficiency virus SIVmac239 replication. J Virol 77: 2736-2740.

5. Brown CR, Czapiga M, Kabat J, Dang Q, Ourmanov I, et al. (2007) Unique pathology in simian immunodeficiency virus-infected rapid progressor macaques is consistent with a pathogenesis distinct from that of classical AIDS. J Virol 81: 5594-5606.

SUPPLEMENTARY TABLES

Table S1. P values for the comparison of SIV viremia, antigenemia and DNA load between IL-7-treated and untreated monkeys, obtained by Wilcoxon rank sum test. Statistically significant p values are bolded.

Day	SIV Viremia	SIV Antigenemia	SIV DNA		
			Blood	GALT	Lymph nodes
**4**	**0.0430**	0.3095	nt	nt	nt
**7**	0.2403	0.3095	nt	nt	nt
**11**	0.2403	0.2403	nt	nt	nt
**14**	0.0823	0.7922	0.9048	0.4000	nt
**18**	0.9307	0.9307	nt	nt	nt
**21**	0.7922	0.9307	nt	nt	nt
**24**	0.4286	0.9307	nt	nt	0.4000
**28**	0.6623	0.6623	nt	nt	nt
**35**	0.4286	0.9307	nt	nt	nt
**41**	0.7922	0.9307	nt	nt	nt
**48**	nt	0.9307	nt	nt	nt
**54**	nt	0.9307	nt	nt	nt
**68**	0.6623	1.0000	nt	nt	nt
**77**	0.7922	1.0000	0.4000	nt	nt

nt = not tested

Table S2. MHC haplotype and baseline peripheral blood $CD3^+$ and $CD4^+$ T-lymphocyte counts in untreated and IL-7-treated macaques.

	MHC haplotype	MHC score*	$CD3^+$ T cells	$CD4^+$ T cells
**Untreated:**				
H743	A01-A08	+2	1831	925
H744	B01	-1	2471	1774
H745	A08	+1	2878	1879
H747	A08-B01	0	2097	1317
H749	n.d.**	0	6771	3656
H753	B08	+1	3194	1996
**Mean (±SD):**		**+3**	**3207±1815**	**1925±938**
**IL-7-treated:**				
H746	B08	+1	2604	1338
H748	A01	+1	3167	2211
H750	A08	+1	3972	2367
H751	B01	-1	3223	2269
H752	n.d.**	0	2767	1486
H754	A02	0	2233	1351
**Mean (±SD):**		**+2**	**2994±603**	**1845±483**

* The MHC score was calculated by attributing a positive score (+1) to alleles associated with protection from SIV disease/viral load and a negative score (-1) to alleles associated with increased viral load/accelerated disease progression; no score was attributed to alleles with no known effects on SIV disease progression.

** n.d. = none detected. The animals were negative for the MHC alleles tested (A01, A02, A08, A11, B01, B03, B04, B08, B17).

SUPPLEMENTARY FIGURES

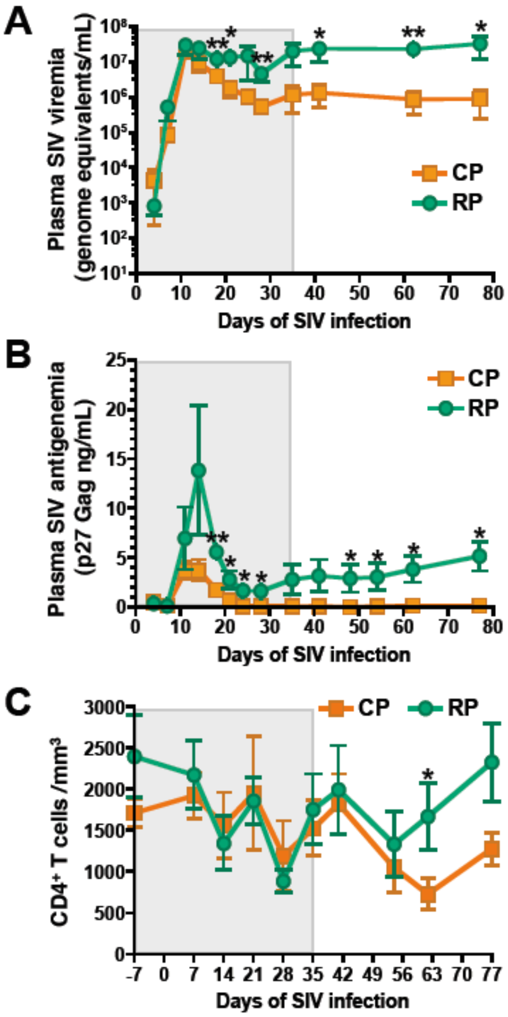

Figure S1. Levels of SIV replication and CD4$^+$ T cells in conventional progressor (CP) versus rapid progressor (RP) SIV-infected macaques. Mean levels (± SEM) of SIV plasma viremia (A) and p27$_{Gag}$ antigenemia (B) in CP (orange) and RP (green). RP animals showed higher levels of both SIV viremia and antigenemia compared to CP at several time points, starting on day 18 post-infection, as indicated by the asterisks (*$p < 0.05$, ** $p < 0.01$, by Wilcoxon rank sum test). (C) Mean absolute numbers (± SEM) of circulating CD4$^+$ T cells in CP (orange) and RP (green). A significant difference was observed on day 62 post-infection, when RP had higher mean levels of circulating CD4$^+$ T cells compared to CP, as indicated by the asterisk ($p < 0.042$, by Wilcoxon rank sum test).

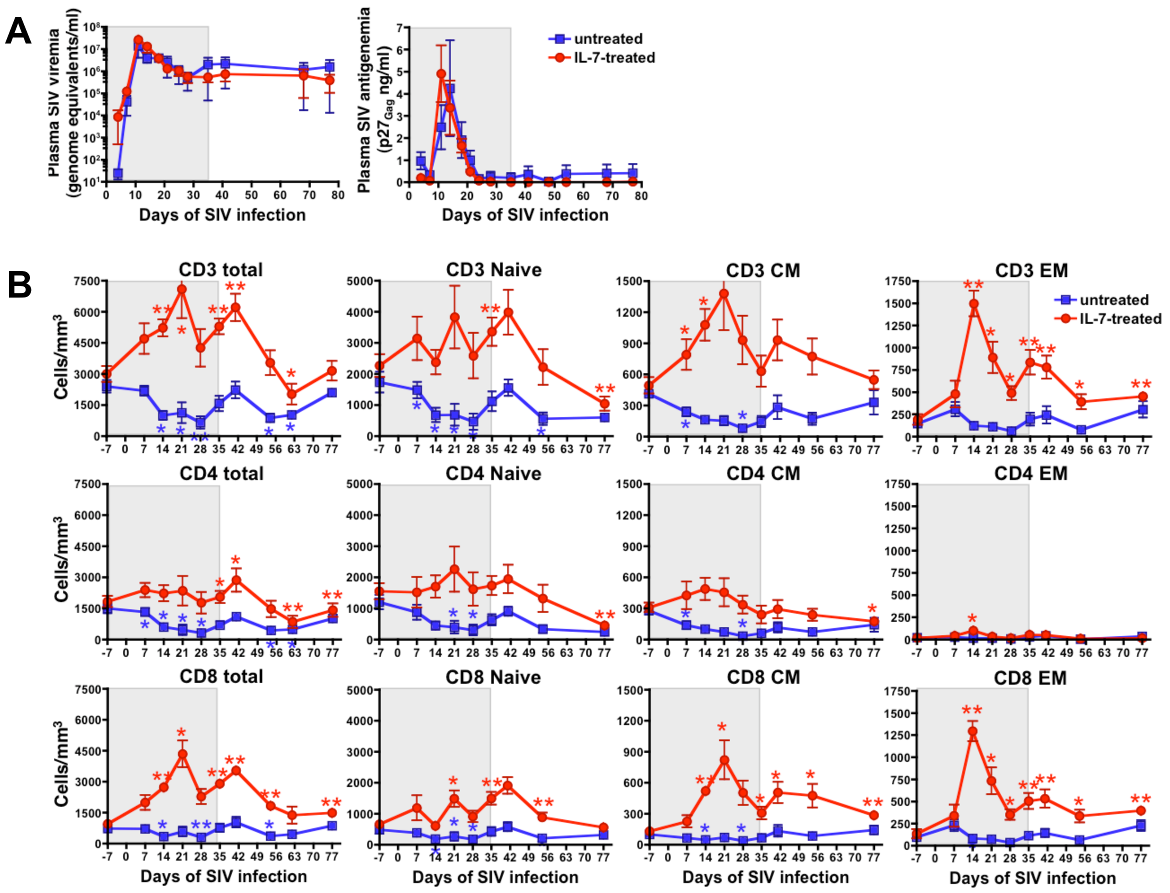

Figure S2. Reanalysis of virological and immunological parameters in IL-7-treated and untreated macaques after exclusion of macaques with rapidly progressive (RP) disease course. (A) Mean levels (± SEM) of SIV plasma viremia and $p27_{Gag}$ antigenemia in untreated (blue) and IL-7-treated (red) animals. (B) Mean absolute numbers (± SEM) of circulating total, naïve, memory and effector $CD4^+$ and $CD8^+$ T cells in untreated (blue) and IL-7-treated (red) animals. The grey shaded area indicates the IL-7-treatment period; blue and red asterisks denote significant differences with baseline values in untreated and IL-7-treated animals, respectively (*p < 0.05, ** p < 0.01, by paired Student's t test).

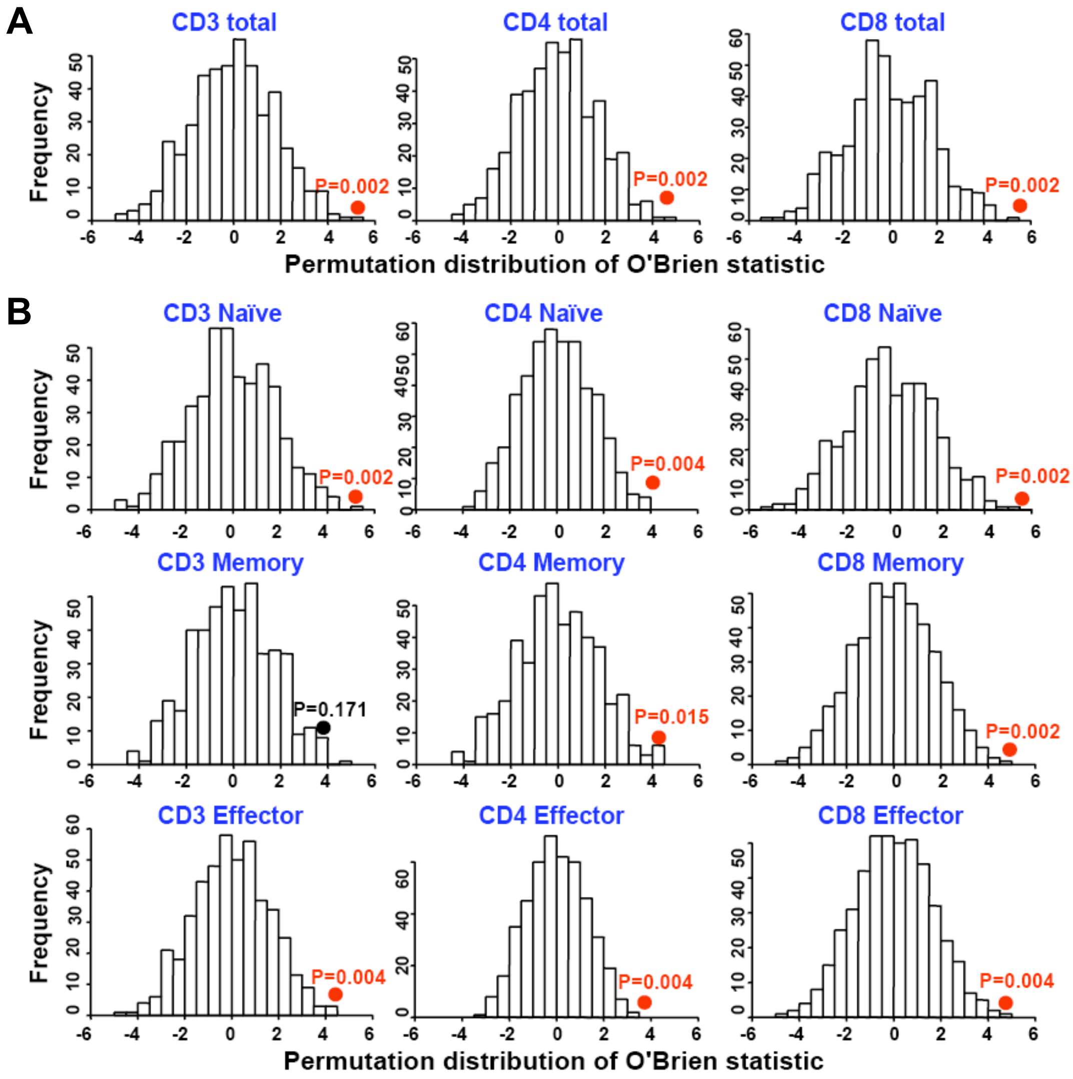

Figure S3. Comparison of changes in peripheral blood T-cell subpopulations between IL-7-treated and untreated macaques during the acute phase of SIV infection. Simultaneous comparisons of changes from baseline to all the time points during acute SIV infection as analyzed by the O'Brien permutation tests. (A) Analyses for total $CD3^+$, $CD4^+$ and $CD8^+$ T cells. (B) Analyses for the naïve, memory and effector T-cell subsets. The histograms represent the distribution of all possible values of the difference between IL-7-treated and untreated animals considering all the possible relabelings of the animals in the two groups (by permutation test); dots indicate the values of the differences between IL-7-treated and untreated animals for the actual trial assignments with the relative p values. Significant differences were observed in all T-cell subsets, except in memory $CD3^+$ T cells.

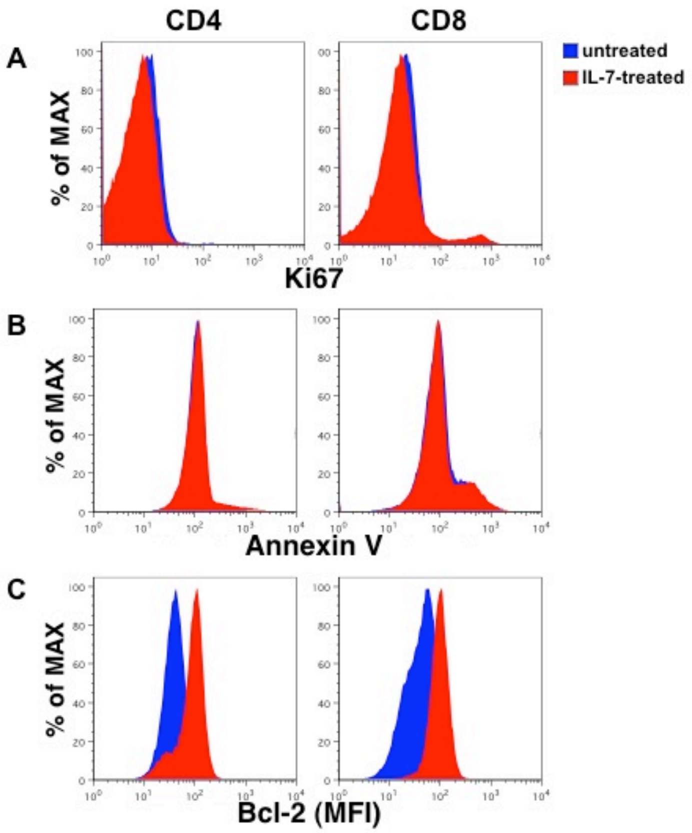

Figure S4. Ki67 expression, Annexin-V binding and Bcl-2 expression in two representative animals (one untreated and one treated with IL-7). Histograms show the levels of Ki67 expression (A), Annexin-V binding (B) and Bcl-2 expression (C) at day 14 post-infection in $CD4^+$ and $CD8^+$ T cells from two representative animals: one from the untreated group (#749, shown in blue) and one from the IL-7-treated group (#746, shown in red).

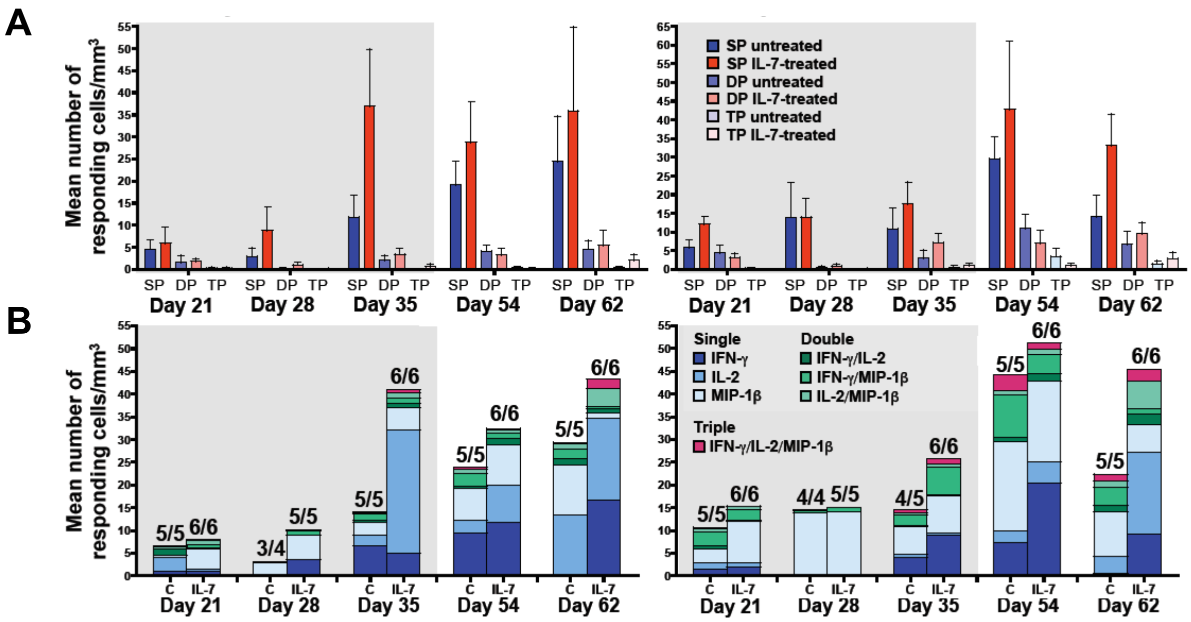

Figure S5. SIV Gag-specific T-cell responses in IL-7-treated and untreated macaques. (A) Mean absolute numbers (± SEM) of $CD4^+$ and $CD8^+$ T cells producing one cytokine (single-producer, SP), two cytokines (double-producer, DP) or all three cytokines (triple-producer, TP) in response to SIV Gag peptide stimulation in untreated (shades of blue) and IL-7-treated (shades of red) animals. (B) Mean absolute numbers of SIV Gag-responding $CD4^+$ and $CD8^+$ T cells in untreated controls (c) and IL-7-treated animals (IL-7). The bars indicate the mean numbers of total responding cells; the colors indicate the mean numbers of IFN-γ, IL-2, or MIP-1β single-producer cells (shades of blue), IFN-γ/IL-2, IFN-γ/MIP-1β or IL-2/MIP-1β double-producer cells (shades of green), and IFN-γ/IL-2/MIP-1β triple-producer cells (purple). The numbers above each bar indicate the fraction of monkeys that gave a measurable response over background to SIV Gag peptides at the corresponding time point. The grey-shaded areas indicate the IL-7-treatment period.

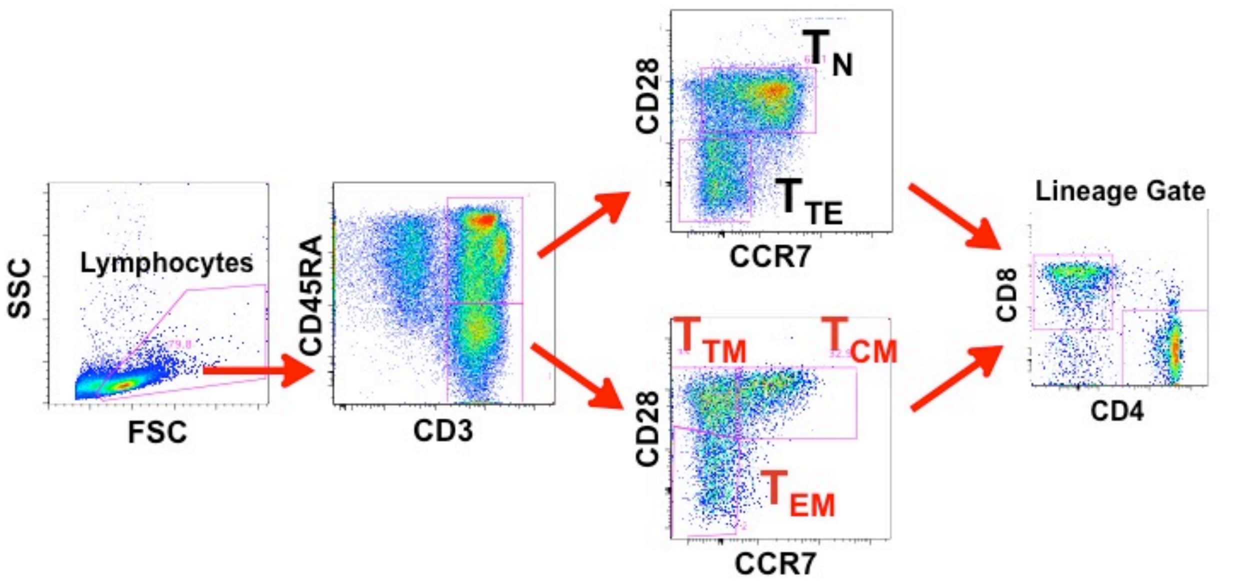

Figure S6. Gating strategy used for the identification of naïve and memory T-cell subsets. Lymphocytes were selected within the lymphocyte gate in a SSC vs FSC dot plot and T cells were further selected for CD3 positivity. Within the $CD3^+$ T-cells gate, $CD45RA^-$ cells were selected and further subdivided into central memory (T_{CM}), transitional memory (T_{TM}) and effector memory (T_{EM}) cells by using a combination of the surface markers CCR7 and CD28 as follows: T_{CM} $CCR7^+$ $CD28^+$, T_{TM} $CCR7^-$ $CD28^+$ and T_{EM} $CCR7^-$ $CD28^-$. Within the $CD45RA^+$ subpopulation, naïve (T_N) and terminal effector (T_{TE}) cells were identified using the same combination of CCR7 and CD28 markers as double positive and double negative cells, respectively. $CD4^+$ and $CD8^+$ T-cell lineages cells were subsequently identified on the gated subpopulations.
